# Cohort profile: design and methods of the Chinese colorectal, breast, lung, liver, and stomach cancer screening trial (C-BLAST)

**DOI:** 10.20892/j.issn.2095-3941.2023.0278

**Published:** 2023-10-27

**Authors:** Yubei Huang, Zhangyan Lyu, Yu Zhang, Xiaomin Liu, Yacong Zhang, Ya Liu, Chao Sheng, Hongyuan Duan, Zeyu Fan, Chenyang Li, Xiao Lin, Zhuowei Feng, Lu Zheng, Zhaoxiang Ye, Hong Lu, Ying Zhu, Dejun Zhou, Xi Wei, Li Ren, Bin Meng, Fangfang Song, Fengju Song, Kexin Chen, the C-BLAST Group

**Affiliations:** 1 Department of Cancer Epidemiology and Biostatistics, Key Laboratory of Molecular Cancer Epidemiology (Tianjin), Tianjin’s Clinical Research Center for Cancer, National Clinical Research Center for Cancer, Tianjin Medical University Cancer Institute and Hospital, Tianjin 300060, China; 2 Department of Radiology, Tianjin Medical University Cancer Institute and Hospital, Tianjin 300060, China; 3 Department of Breast Imaging Diagnosis, Tianjin Medical University Cancer Institute and Hospital, Tianjin 300060, China; 4 Department of Endoscopy, Tianjin Medical University Cancer Institute and Hospital, Tianjin 300060, China; 5 Department of Ultrasound Imaging Diagnosis, Tianjin Medical University Cancer Institute and Hospital, Tianjin 300060, China; 6 Department of Clinical Laboratory, Tianjin Medical University Cancer Institute and Hospital, Tianjin 300060, China; 7 Department of Pathology, Tianjin Medical University Cancer Institute and Hospital, Tianjin 300060, China

Given the rapid changes in social structure (urbanization), economic structure (industrialization), and demographic structure (population aging) in China, cancer has become a major public health problem^1^. Extensive evidence has indicated that screening can decrease cancer mortality, particularly among high-risk groups, and several representative national and regional cancer screening programs have been launched in China to cope with the increasing burden of cancer; however, most of the previous studies screened for one type of cancer at a time (single cancer screening mode), while few focused on combined screening for several types of cancer (combined cancer screening mode) in the general population. Given the rising burden of multiple cancers worldwide, it is still unclear whether the combined cancer screening mode can improve screening effectiveness compared to the single cancer screening mode. Moreover, it is unclear if the combined cancer screening mode can yield more benefits than the traditional screening mode^2–5^ after integrating new pan-cancer markers and rapidly developing artificial intelligence (AI) technology, especially in countries and regions with limited healthcare resources. To assess the feasibility and effectiveness of the combined cancer screening mode in China, we launched the Chinese Colorectal, Breast, Lung, Liver, And Stomach cancers Screening Trial (C-BLAST) in 2017 in Tianjin City, China. This article is aimed at describing the overall design of the C-BLAST.

## Overall design of C-BLAST

**Figure 1:**
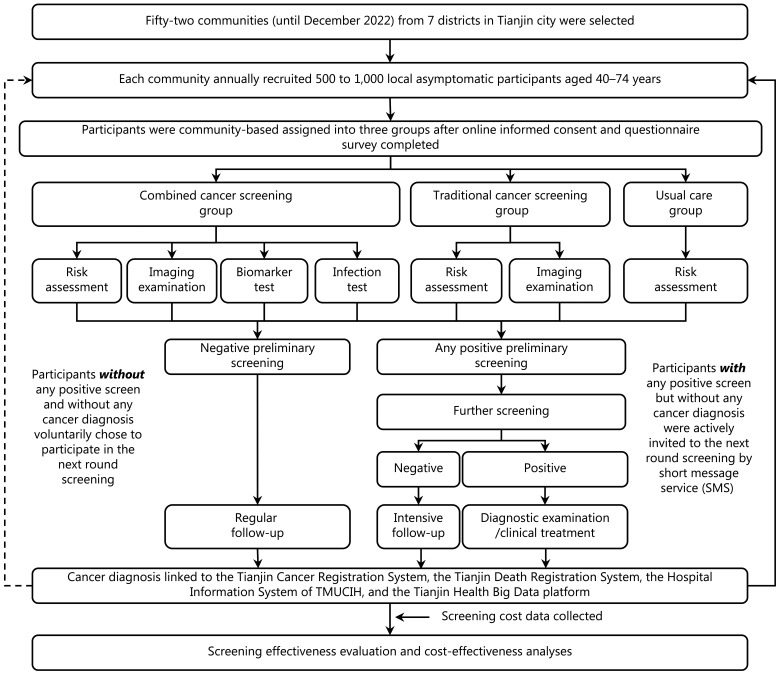
Flowchart of participant selection and intervention assignments in the C-BLAST. SMS, short message service; TMUCIH, Tianjin Medical University Cancer Institute and Hospital.

The C-BLAST is an open multi-stage, controlled intervention trial designed to recruit > 200,000 asymptomatic participants 40–74 years of age from Tianjin City, China, with the goal to cover nearly 50% of the local targeted population. After online informed consent was obtained, all asymptomatic participants 40–74 years of age in each community were assigned to the same intervention group, while different communities in the same district were randomly assigned to different intervention groups. To ensure adequate numbers of cases detected in the usual care group (the detection rate is expected to be less than one-third of the combined cancer screening group) and to ensure comparisons of different screening combinations in the combined cancer screening group, the participants in the usual care, traditional cancer screening, and combined cancer screening groups were assigned at an approximate ratio of 7:1:2. All participants underwent community hospital-based preliminary screening and participants with positive preliminary screening received further tertiary hospital-based screening. Participants in the usual care group only received a questionnaire-based cancer risk assessment during preliminary screening. Participants in the traditional screening group with a high risk of cancer after risk assessment underwent an imaging examination, as recommended by current guidelines. Participants in the combined screening group received a cancer risk assessment, imaging examination, and biomarker and infection tests (**Figure 1**). Both C-BLAST and the extended studies were approved by the Institutional Review Board of Tianjin Medical University Cancer Institute and Hospital (TMUCIH).

## Community and population selection

**Figure 2:**
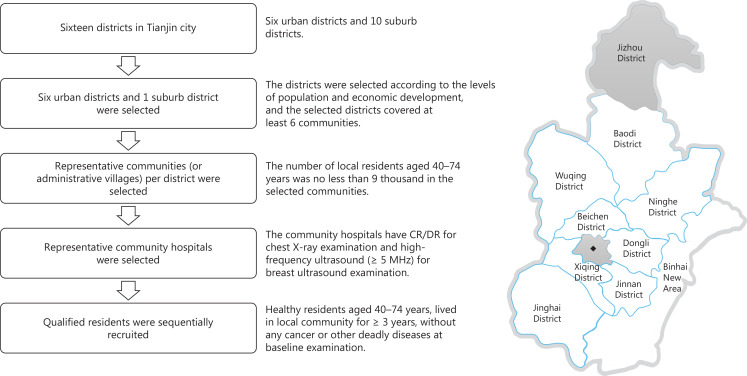
Flowchart of district and community selection in the C-BLAST. CR/DR, computed radiography or digital radiography; ◆, including Heping, Hexi, Hedong, Hebei, Nankai, and Hongqiao districts.

Sixteen districts and counties, and nearly 5,690 communities or administrative villages in Tianjin City were identified in the Tianjin Statistics Yearbook 2017. We selected six urban districts (Heping, Hexi, Hedong, Hebei, Nankai, and Hongqiao) and one suburban district (Jizhou) to obtain a relatively representative sample, according to the population size and level of economic development. The selected districts covered at least six communities and at least two communities per intervention group. Representative communities were selected according to the following inclusion criteria: 1. at least 9,000 local residents between 40 and 74 years of age in the selected community; and 2. availability of computed or digital radiography for chest X-ray examination and high-frequency ultrasound with standard linear frequency probes ≥ 5 MHz for breast ultrasound examination at community hospitals (**Figure 2**). Two additional criteria were required for communities assigned to the combined screening group: 1. availability of C14 urea breath tests to detect the presence of *Helicobacter pylori*; and 2. availability of a refrigerator maintained at 4°C to temporarily store at least 30 blood samples.

Each selected community recruited 500–1,000 healthy residents annually according to the following inclusion criteria: 1. 40–74 years of age; 2. living in the local community for > 3 years; 3. no evidence of cancer before recruitment; and 4. no other severe diseases at baseline, including arrhythmias, myocardial infarction, heart or liver failure, hemorrhagic tendency, or acute perforation of the esophagus, stomach, or duodenum.

## Screening platform

**Figure 3:**
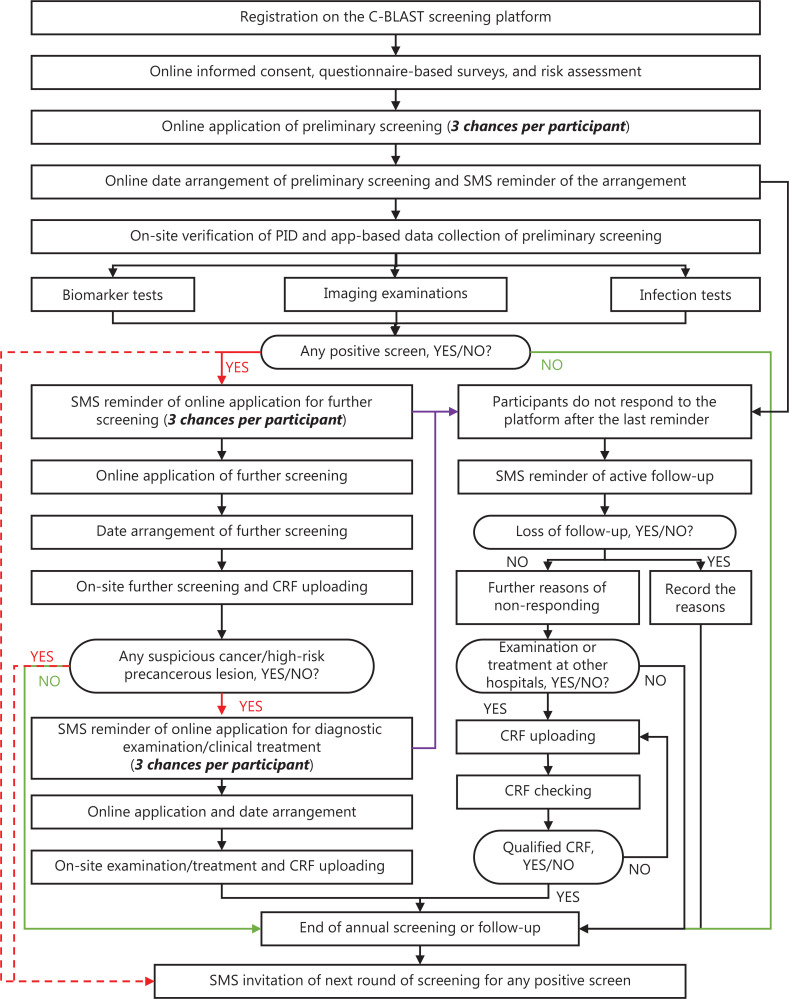
Flowchart of the C-BLAST screening platform. SMS, short message service; PID, personal identification; CRF, case report form.

After registering on the C-BLAST screening platform (https://c-blast.tjmucih.cn:10443/tjzl/), qualified participants were required to sign informed consent forms online and complete online questionnaire-based surveys, including a baseline questionnaire, a questionnaire on knowledge and attitudes toward cancer screening, and the General Mental Health 12-item Scale [GHQ-12 (data collected since 2023)]^6^. The C-BLAST screening platform provided cancer risk assessment and personalized health recommendations for cancer prevention after the questionnaire-based surveys were completed. Moreover, the C-BLAST screening platform provided abundant online guidance and a short message service (SMS) to help the participants complete the two-step screening, improve the efficiency of the screening procedure, and decrease potential loss to follow-up. The detailed screening procedure is shown in **Figure 3**.

## Questionnaire survey and risk assessment

After online informed consent, qualified participants were required to complete an online baseline questionnaire survey to collect participant-reported information, including demographics, anthropometric measurements (height, weight, waist circumference, and hip circumference), family history of cancer among first-, second-, and third-degree relatives, history of cancer-related diseases, history of cancer screening, history of medication (nutrient and supplement intake), cigarette smoking, alcohol consumption, physical activity, diet, environmental and occupational exposures, infections, psychological factors, and female-specific information (age at menarche, age at menopause, fertility, breast feeding, and abortion).

Although several well-performing risk prediction models have been developed for several common cancers, few prediction models have been widely used in real-world cancer intervention studies, including the well-known Gail risk prediction model for breast cancer^7^. Even though the physicians received 3 months of intensive training, no apparent increase was observed in breast cancer risk assessment for women by primary care physicians^8^. Complex risk weights and model algorithms are considered the most important barriers to the application and promotion of these models^8^. Therefore, to simplify the preliminary cancer risk assessment tool, a simple, standardized scoring system was developed based on the number and level of risk factors, including pan-cancer and cancer-specific risk factors, rather than complex risk weights. These risk factors were primarily chosen based on the Cancer Prevention Recommendations from the World Cancer Research Fund/American Institute for Cancer Research and the annual Cancer Progress Report from the American Association for Cancer Research^9,10^. After further excluding factors weakly associated with cancer one-by-one, the optimized tool was determined as the model with similar accuracy as the full model, but with a fewer number of factors. To reduce potential false-positive results, the cancer risk assessment was not directly used to recommend further screening since 2019 but was only used for invitations to participate in the next round of screening.

## Preliminary screening

After the online application for preliminary screening was approved by community physicians, participants underwent preliminary screening at the community hospital on the scheduled date. Before the preliminary screening, a short face-to-face interview was conducted by community physicians with a portable Android device to verify personal identification (PID) by linking to registry information from the screening platform. Both case report forms (CRFs) and original images of preliminary screening were collected on a portable Android device, then submitted to the screening platform.

Community radiologists or sonographers with at least 5 years of experience performed the preliminary imaging examinations, including chest X-ray, breast ultrasound (women only), and liver ultrasound, according to the pre-defined guidelines. Ten milliliters of blood in 2 tubes (4 and 6 mL) was collected from each participant according to the pre-defined guidelines. The 4-mL blood samples were used for routine marker testing, including routine tumor markers, complete blood count, fasting blood glucose, and lipid profile, while the 6-mL blood samples were centrifuged and the DNA was extracted and stored at −80°C until further testing for genome-wide association analysis, circulating cell-free DNA, and other new genetic markers. The C14 urea breath test was used to detect current *H. pylori* infection, whereas Epstein-Barr virus (EBV) and hepatitis B virus (HBV) infection status was determined by testing for early IgG antibody to EBV (EBV-EA-IgG) and HBV surface antigen (HBsAg), respectively.

Positive preliminary screening was defined as any abnormal or suspicious screening finding on chest X-ray (any non-calcified nodule or mass with a diameter ≥ 6 mm, any other abnormality indicating suspicion for lung cancer), Breast Imaging Reporting & Data System (BI-RADS) 4 or 5 on preliminary breast ultrasound, potential cirrhotic liver nodules or any other abnormalities in preliminary liver ultrasound, any routine marker above the reference value, or any positive infection (C14 urea breath test ≥ 1000 dpm or positive HBsAg).

## Further screening

For participants with positive preliminary screening findings, the screening platform automatically reminded the participants of the online application for further screening through a SMS. The further screening examinations included lung low-dose computed tomography (LDCT) screening for participants at high risk for lung cancer, breast ultrasonography and mammography for breast cancer, liver ultrasound for liver cancer, and upper gastrointestinal endoscopy for stomach cancer. To improve the data quality in further screening, all further screening examinations were performed by radiologists, sonographers, or endoscopists with at least 5 years of experience at TMUCIH and recorded with online CRFs according to pre-defined guidelines. The original images were also required to be uploaded within 1 month according to the criteria for the preliminary original images.

Notably, colorectal cancer screening has been covered by the Tianjin Colorectal Cancer Screening Program, a basic public health program in Tianjin covering a large percentage of the population. To avoid the duplication of resources for colorectal cancer screening, all data on colorectal cancer screening, including the fecal immunochemical test and colonoscopy examination findings, were obtained by linking to the colorectal cancer screening system.

A positive further screening finding was defined as Lung CT Screening Reporting & Data System (LUNG-RADS) 4A/4B/4X on LDCT, BI-RARDS 4/5 on further breast ultrasonography or mammography, potential cirrhotic liver nodules or Liver Reporting & Data System (LI-RADS) 3 on further liver ultrasound, or any abnormal/suspicious precancerous lesions (moderate–to–severe atrophic gastritis, intestinal metaplasia, or dysplasia) on biopsy after upper gastrointestinal endoscopy.

## Follow-up and endpoints

Patients with suspected cancer based on positive findings on further screening were recommended to undergo a diagnostic examination and clinical treatment. Active follow-up was performed to confirm whether death occurred and/or a cancer diagnosis was given, or to determine the reasons for non-response to the screening platform after the last SMS reminder following a positive screening finding. Participants with a positive screening finding, including high risk scores after cancer risk assessment but without any confirmed cancer, were invited to participate in the next round of screening. In addition, annual passive follow-up was performed for all participants by linking to the local Cancer Registration System, the local Death Registration System, the Hospital Information System (HIS) of TMUCIH, and the local Health Big Data platform associated with the HISs of 96 secondary and tertiary hospitals in Tianjin.

Incident cancer was confirmed by pathologic evaluation, clinical diagnosis, the 10^th^ revision of the International Statistical Classification of Diseases and Related Health Problems (ICD-10) code indicating cancer or linking to any local health big-data system. If a cancer diagnosis was confirmed, further clinical information was collected, including the date of diagnosis, tumor size, lymph node metastasis, distal metastasis, and available treatment data. Cancer-specific deaths were confirmed by clinical diagnosis, ICD-10 codes indicating cancer deaths, or linking to health big-data systems.

All participants were censored at the date of incident cancer or death, loss to follow-up, or the latest qualified follow-up, whichever came first. Until December 2022, the C-BLAST had recruited 153,475 participants with 184,058 qualified screenings, including 30,583 repeated screenings among 24,772 participants, and 2901 participants with any cite of cancers before recruitment (3427 registrations), and a total of 2016 participants died before the latest follow-up.

## Discussion

Combined cancer screening is expected to decrease overlap among basic labor output, devices, and costs of various cancer screening efforts compared to the single cancer screening mode, thereby increasing the cost-effectiveness of the single cancer screening mode. More importantly, owing to shared risk factors^11^ and potential shared pathogenic genetic mutations^12,13^, combined cancer screening would potentially decrease missed early detection for multiple cancers and would combine the weak benefits of single cancer screening to achieve clear pooled benefits due to an increased adoption of healthful lifestyles (smoking cessation, alcohol abstinence, exercise, weight control, and healthy diet) after multiple positive screening findings and health-related advice^14,15^. Furthermore, the adoption of healthy lifestyles would also prevent more deaths from vascular and respiratory conditions than from cancer given the multimorbidity of non-communicable diseases^16–18^. Beyond the above benefits, multiple positive screens would increase active seeking of additional healthcare, thereby potentially increasing over-examination, over-diagnosis, and the psychological stress resulting from cancer diagnosis^19,20^. Therefore, to evaluate the comprehensive effectiveness of combined cancer screening, researchers should shift their focus from screening targeted cancers to all-site neoplasms, from assessing cancer-specific mortality to all-cancer mortality, from effectiveness evaluation based on endpoints to promotion of healthy lifestyles, and from examining benefits alone to benefit-harm trade-offs.

The combined cancer screening design, particularly the use of multiple screening tests and examinations in the selected population (risk assessment, imaging examination, biomarker tests, infection tests, and original image collection) will enable the C-BLAST to investigate more novel pan-cancer markers and more optimized combined screening strategies compared to previous studies. Moreover, the collected original images will enable the C-BLAST to develop more AI-assisted screening diagnosis tools and provide more comprehensive cross-omics analyses after integrating the exposome, genomics, and radiomics^2–4^. The annually updated GHQ-12 data will allow for evaluation of the short- and long-term effects of positive screening results on psychological stress, screening behavior, and cancer incidence^19,20^. The annually updated investigation of knowledge and attitudes toward cancer screening would enable C-BLAST to evaluate whether adherence to personalized health recommendations after cancer risk assessment interacts with cancer screening and increasing cancer prevention benefits. Finally, the screening platform can be used as a model for a comprehensive platform for collecting screening data at different levels and integrating AI analysis, data sharing, and health education.

In conclusion, the C-BLAST is a community-based intervention trial based on an open cohort, aimed at evaluating the real-world feasibility and efficacy of combined cancer screening, improving the overall capacity for cancer screening in low-resource areas, and finally significantly decreasing the burden of major types of cancers. The C-BLAST is also expected to provide insight into combined cancer risk assessment, pan-cancer biomarker identification, AI-assisted cancer screening, and comprehensive interventions for multimorbid non-communicable diseases.
